# Facile Synthesis, Characterization of Poly-2-mercapto-1,3,4-thiadiazole Nanoparticles for Rapid Removal of Mercury and Silver Ions from Aqueous Solutions

**DOI:** 10.3390/polym10020150

**Published:** 2018-02-06

**Authors:** Shaojun Huang, Chengzhang Ma, Chao Li, Chungang Min, Ping Du, Yi Xia, Chaofen Yang, Qiuling Huang

**Affiliations:** 1 Research Center for Analysis and Measurement, Kunming University of Science and Technology, Kunming 650093, China; minchungang@163.com (C.M.); dupin515@163.com (P.D.); xiayi0125@163.com (Y.X.); yangmlh@163.com (C.Y.); hql1975@eyou.com (Q.H.); 2 School of Materials Science and Engineering, Kunming University of Science and Technology, Kunming 650093, China; mcz219@yeah.net (C.M.); lichao2527@yeah.net (C.L.)

**Keywords:** poly-2-mercapto-1,3,4-thiadiazole, synthesis, characterization, nanoparticles, adsorption, heavy metal ions, mercury, silver

## Abstract

Industrial pollution by heavy metal ions such as Hg^2+^ and Ag^+^ is a universal problem owing to the toxicity of heavy metals. In this study, a novel nano-adsorbent, i.e., poly-2-mercapto-1,3,4-thiadiazole (PTT), was synthesized and used to selectively adsorb mercury and silver ions from aqueous solutions. PTT nanoparticles were synthesized via chemical oxidative dehydrogenation polymerization under mild conditions. Oxidant species, medium, monomer concentration, oxidant/monomer molar ratio, and polymerization temperature were optimized to obtain optimum yields. The molecular structure and morphology of the nanoparticles were analyzed by ultraviolet-visible (UV-Vis), Fourier transform infrared (FT-IR), matrix-assisted laser desorption/ionization/time-of-flight (MALDI/TOF) mass and X-ray photoelectron (XPS) spectroscopies, wide-angle X-ray diffraction (WAXD), theoretical calculations and transmission electron microscopy (TEM), respectively. It was found that the polymerization of 2-mercapto-1,3,4-thiodiazole occurs through head-to-tail coupling between the S(2) and C(5) positions. The PTT nanoparticles having a peculiar synergic combination of four kinds of active groups, S–, –SH, N–N, and =N– with a small particle size of 30–200 nm exhibit ultrarapid initial adsorption rates of 1500 mg(Hg)·g^−1^·h^−1^ and 5364 mg(Ag)·g^−1^·h^−1^ and high adsorption capacities of up to 186.9 mg(Hg)·g^−1^ and 193.1 mg(Ag)·g^−1^, becoming ultrafast chelate nanosorbents with high adsorption capacities. Kinetic study indicates that the adsorption of Hg^2+^ and Ag^+^ follows the pseudo-second-order model, suggesting a chemical adsorption as the rate-limiting step during the adsorption process. The Hg^2+^ and Ag^+^-loaded PTT nanoparticles could be effectively regenerated with 0.1 mol·L^−1^ EDTA or 1 mol·L^−1^ HNO_3_ without significantly losing their adsorption capacities even after five adsorption–desorption cycles. With these impressive properties, PTT nanoparticles are very promising materials in the fields of water-treatment and precious metals recovery.

## 1. Introduction

Over the past few decades, heterocyclic and heterochain aromatic polymers have received considerable attention because of their wide use in the fields of electrode materials, sensors, adsorbents, cathode materials for rechargeable lithium-ion batteries and so on [1,2,3,4,5]. As a new kind of functional aromatic heterocyclic polymer, the 1,3,4-thiadiazole-ring containing polymers have revealed a promising future in the fields of rechargeable lithium batteries, biological and chemical sensors, clinical diagnosis and pharmacological studies, optoelectronic devices, and heavy-metal-ion adsorbents because of their unique energy storage performance, electrocatalytic activity, and electron-rich properties [6,7,8]. The 1,3,4-thiadiazole-ring containing polymers can be synthesized either by an electrochemical oxidative method [9,10,11] or by a chemical oxidative method [8,12,13]. Several typical 1,3,4-thiadiazole-ring containing polymers, such as poly-2-amino-1,3,4-thiadiazole (PAT), poly-2,5-dimercapto-1,3,4-thiadiazole (PBT), and poly-2-amino-5-mercapto-1,3,4-thiadiazole (PAMT) have been successfully prepared via electrochemical synthesis in 0.10 mol·L^−1^ H_2_SO_4_ or phosphate buffer solution [9,10,11]. However, the chemical oxidative synthesis of these polymers is still very challenging because 1,3,4-thiadiazole-ring containing monomers can easily bind metal salt oxidants to generate a complex or metal coordination polymer [14,15,16,17] or an open-loop reaction of the 1,3,4-thiadiazole-ring containing monomers may be induced with a very strong oxidizing agent. Apparently, the synthesis of 1,3,4-thiadiazole-ring containing polymers is much more difficult than that of traditional polymers such as polyaniline, polypyrrole, and polythiophene. Fortunately, PBT polymers have been successfully synthesized through a chemical oxidative polymerization method in methanol, water-methanol (1:1, *v/v*), water-ethanol (1:2, *v/v*), or 0.1 mol·L^−1^ acetic acid-sodium acetate aqueous solution with iodine, hydrogen peroxide, or ammonium persulfate as an oxidant. To the best of our knowledge, there is no report regarding the synthesis of poly-2-mercapto-1,3,4-thiadiazole (PTT) as yet.

On the other hand, the water pollution caused by human activities has become a major global concern. Mercury ion, as one of the most toxic heavy metal ions usually detected in aquatic systems, can cause a series of serious human diseases such as paralysis, serious intestinal and urinary syndrome, central nervous system dysfunction and even worse, death, even at quite low concentrations [18]. Many sources including chloralkali plants, oil refineries, power plants, paper and pulp factories, rubber processing plants, fertilizer production plants, and similar industries are responsible for mercury discharge into the environment [19,20]. Silver, as both a heavy metal and a precious metal, also has some toxicity to humans and mammals. Frequent exposure to silver ions can be very harmful to health, possibly leading to various disorders and diseases, such as hypertension, behavioral changes, oxidative stress, etc. [21]. Various industries, including photography, electroplating, medicine, secondary battery, and metallurgy, may be responsible for the release of a thousand tons of silver-containing compounds annually into waste water streams [22,23,24].

Though various techniques have been applied to treat Hg^2+^- and Ag^+^-contaminated waters, adsorption is still one of the popular ways that mercury and silver can be cheaply and easily removed [25]. Previous studies have found that Hg^2+^ and Ag^+^ ions show a very strong affinity to N- and S-containing functional groups, such as –NH_2_, =N–, –C≡N, –SR, –SH, etc. [26,27,28,29] Many effective Hg^2+^- and Ag^+^-adsorbents have been prepared by means of immobilizing these groups onto the surface of various solid matrixes involving activated carbon [30], silica [21,26,31], polymers [32,33,34], and biomass [35,36]. However, there are some disadvantages in the field of the adsorption of Hg^2+^ and Ag^+^, such as low adsorption capacity, low adsorption rate, poor selectivity, and low repeated utilization ratio. Polymer adsorbents have recently attracted strong attention because of their large sorption capacity, easy preparation, high efficiency, and regeneration ability [25]. Nevertheless, the development of new polymer adsorbents for rapid, selective, and effective removal of heavy metals and recovery of precious metals is still a great challenge for both industrial wastewater treatment and environmental remediation. Sulfur and nitrogen-rich PTT polymers are promising adsorbents for Hg^2+^ and Ag^+^ because of the multi-functional groups on their molecular chains, such as –S–, –SH, N–N, and =N– groups.

In this work, PTT polymers were synthesized by using chemical oxidative dehydrogenation polymerization of TT monomers. The effects of synthetic parameters including oxidant species, polymerization medium, TT monomer concentration, oxidant/TT molar ratio, and polymerization temperature on the yields of PTTs were investigated and optimized. The chemical structures and micro-morphologies were studied by ultraviolet-visible (UV-Vis), Fourier transform infrared (FT-IR), matrix-assisted laser desorption/ionization/time-of-flight (MALDI/TOF) mass and X-ray photoelectron (XPS) spectroscopies, wide-angle X-ray diffraction (WAXD), theoretical calculations and transmission electron microscopy (TEM), respectively. Finally, the adsorption performance for heavy metal ions and regeneration of the polymer were studied in detail.

## 2. Experimental Section

### 2.1. Chemicals

The chemicals 2-Mercapto-1,3,4-thiadiazole (1,3,4-thiadiazole-2-thiol, TT), iodine (I_2_), sodium hypochlorite (NaClO), benzoyl peroxide (BPO), hydrogen peroxide (H_2_O_2_), ammonium persulfate [(NH_4_)_2_S_2_O_8_], anhydrous copper chloride (CuCl_2_), *N*,*N*-dimethylformamide (DMF), methanol (CH_3_OH), anhydrous ethanol (C_2_H_5_OH), hydrochloric acid (HCl), nitric acid (HNO_3_), ethylenediaminetetra-acetic acid (EDTA) disodium salt, Hg(NO_3_)_2_, Pb(NO_3_)_2_, AgNO_3_, Ni(NO_3_)_2_, Co(NO_3_)_2_, Cr(NO_3_)_3_, Cd(NO_3_)_2_, Fe(NO_3_)_3_, Zn(NO_3_)_2_, Cu(NO_3_)_2_, dithranol (DIT), and other chemicals were purchased from the Chemical Reagent Corp. in Kunming, China. All reagents were of analytical grade and used without further purification.

### 2.2. Synthesis of PTT Nanoparticles

In a typical synthesis of PTT polymer, 0.5991 g (5 mmol) of 2-mercapto-1,3,4-thiadiazole (1,3,4-thiadiazole-2-thiol, TT) was dissolved in 3 mL of DMF with efficient stirring to give a colorless monomer solution. Then, 0.6722 g (5 mmol) of anhydrous copper chloride was added in 10 mL of DMF, and the mixture was stirred for 10 min to give a yellow green oxidant solution. Next, the DMF solution of the oxidant was added dropwise to the TT monomer solution, and the reactants were allowed to react at 25 °C for 24 h with magnetic stirring. Upon adding oxidant solution drop by drop, the monomer solution changed in color from colorless to reddish brown, and finally to black. After completion of the reaction, 200 mL of 0.047 mol·L^−1^ aqueous EDTA solution was added into the reaction mixture to precipitate the polymer, producing a large amount of green precipitate. The precipitate was washed with sufficient aqueous EDTA solution (0.047 mol·L^−1^), deionized water, and ethanol until the supernatant was colorless or light colored with the purpose of removal of the remaining oxidant copper chloride, by-product cuprous chloride, residual monomers, and oligomers. Lastly, the precipitate was dried at 80 °C for 24 h in air to give yellow polymer nanoparticles with a yield of 72%.

### 2.3. Adsorption Experiments

The adsorption of heavy metal ions onto PTT polymers was conducted in a batch experiment. It was carried out in a series of 100 mL conical flasks containing 50 mg of the PTT particles and 50 mL of heavy metal ion aqueous solution whose initial concentration ranged from 20 to 200 mg·L^−1^. The conical flasks were sealed and shaken in a water bath (25 °C, 200 rpm). After shaking for the desired period, the mixture was centrifuged at a rotation speed of 3800 rpm for 20 min to give a supernatant and a precipitate. The concentration of heavy metal ion in the supernatant was analyzed using inductively coupled plasma atomic emission spectrometry (ICP-AES), and the precipitate was dried to constant weight at 80 °C for further analysis. The selectivity adsorption of the PTT for a certain kind of heavy metal ion was similar to the above procedure, except the aqueous solution contained a variety of metal ions. The adsorption capacity and the adsorption ratio of the PTT adsorbent were calculated according to the equations in the previous literature [37].

### 2.4. Desorption Experiments

For desorption studies, 0.2 g of PTT nanoparticles was loaded with metal ions (Ag^+^ and Hg^2+^) using 100 mL of 200 mg·L^−1^ metal ion solution at 25 °C for 2 h. The agitation rate was fixed at 200 rpm. After adsorption, the metal ion-loaded PTT nanoparticles were separated by centrifugation and gently washed with deionized water to remove any non-adsorbed metal ions. The nanoparticles were then agitated with 100 mL of eluent (0.1 mol·L^−1^ EDTA or 1 mol·L^−1^ HNO_3_). The final concentration of metal ions in the aqueous phase was determined by using ICP-AES. The desorption ratio of metal ions from PTT nanoparticles was calculated as the amount of metal ions in the desorption eluent divided by the amount of metal ions adsorbed on PTT. To examine the reusability of the nanoparticles, this adsorption–desorption cycle was repeated five times by using the same affinity adsorbent.

### 2.5. Characterization and Measurements

The UV-Vis spectra of the monomer and polymer solutions or dispersions in DMF were recorded using a TU-1901 UV-Vis double-beam spectrophotometer made by Beijing Purkinje General Instrument Co., Ltd. (Beijing, China) in a wavelength range from 190 to 900 nm at a scanning speed of 400 nm·min^−1^. Fourier transform infrared (FT-IR) spectra for PTT/KBr pellets were recorded on a Bruker TENSOR 27 FT-IR spectrometer (Bruker, Ettlingen, Germany) scanning from 4000 to 400 cm^−1^ with a resolution of 4 cm^−1^ in a transmission mode. Wide-angle X-ray diffraction (WAXD) patterns for powder samples of the PTT polymers and TT monomer were recorded with a Rigaku International Corporation D/max 2000 X-ray diffractometer (Tokyo, Japan) with Cu Kα radiation in a Bragg angle range of 4–90° at a scan rate of 10°·min^−1^. The matrix-assisted laser desorption/ionization/time-of-flight (MALDI/TOF) mass spectra of the tetrahydrofuran (THF) soluble part of PTT were recorded with a Bruker Daltonik GmbH autoflex speed TOF/TOF mass spectrometer (Bruker Corporation, Billerica, MA, USA) using dithranol (DIT) as the matrix. The XPS measurements were carried out with a PHI5000 Versaprobe-II multifunctional scanning and imaging photoelectron spectrometer (Physical Electronics Inc., Chigasaki, Japan) equipped with an Al Kα X-ray source. The surface morphology of the PTT nanoparticles was visualized using a FEI Tecnai G2 TF30 S-Twin field emission TEM (FEI Company, Eindhoven, The Netherlands). The sample suspension in alcohol was dropped onto copper mesh and then dried under an infrared lamp (TEM) prior to the observation. The atomic emission spectra of metal ions in water samples were obtained with a Prodigy High Dispersion ICP–AES (Teledyne Leeman Labs, Hudson, NH, USA).

## 3. Results and Discussion

### 3.1. Synthesis of PTT Nanoparticles

#### 3.1.1. Selection of Oxidant Species

The standard reduction potential (RP) of oxidants exerts a critical effect on the chemical oxidation polymerization. We chose the oxidants with different RPs such as (NH_4_)_2_S_2_O_8_ (RP 2.01 V), BPO, H_2_O_2_ (RP 1.77 V), NaClO (RP 1.49 V), CuCl_2_ (RP 0.86 V), and iodine (I_2_, RP 0.545 V), to polymerize the TT monomers in DMF. It was found that only CuCl_2_ with an RP of 0.86 V could be used as the oxidant to synthesize successfully the poly-2-mercapto-1,3,4-thiadiazole (PTT) polymer. This indicates that the polymerization reaction of TT monomers may take place only if the appropriate oxidant with a moderate RP is used, and oxidants with either higher RP or lower RP are unsuitable for the polymerization.

#### 3.1.2. Screening of Polymerization Medium

**Scheme 1:**
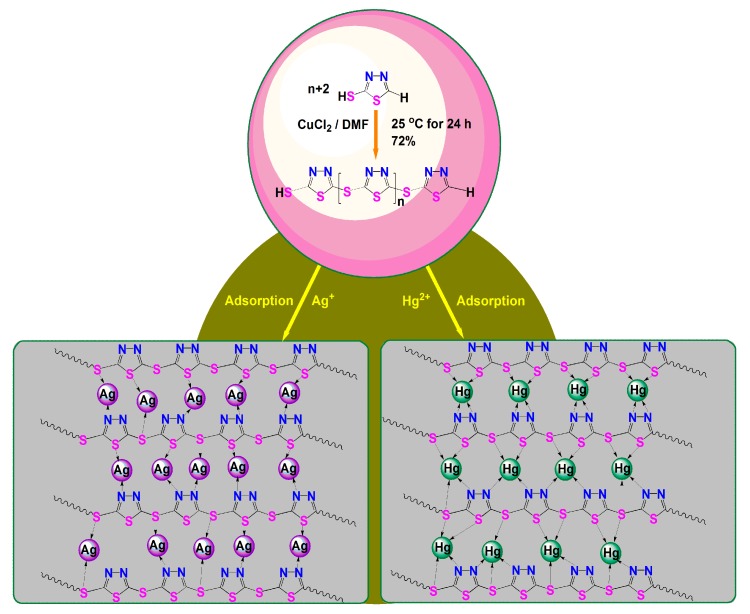
Chemical oxidative dehydrogenation polymerization of 2-mercapto-1,3,4-thiadiazole (TT) and the proposed adsorption mechanism of poly-2-mercapto-1,3,4-thiadiazole (PTT) nanoparticles for Ag^+^ and Hg^2+^.

It was found from a solubility experiment that TT monomer could be dissolved well in DMF, 1 mol·L^−1^ HCl, methanol and ethanol, but only slightly dissolved in pure water. The anhydrous CuCl_2_ oxidant, however, could be dissolved well in DMF and pure water and only partially dissolved in 1 mol·L^−1^ HCl, methanol, and ethanol. So DMF was selected as the reaction medium. On dropping the CuCl_2_ solution into the organic solution of the TT monomer, it gradually became darker in color, changing from colorless to reddish brown and finally to black. This is an indication of the occurrence of the polymerization of TT monomers (Scheme 1). Thus, DMF was chosen as the reaction medium for the subsequent synthesis.

#### 3.1.3. Optimization of TT Monomer Concentration

The effect of monomer concentrations on the yields of the PTT is not considerable. When the TT concentrations increased from 0.27 to 0.33 and 0.38 mol·L^−1^, the yields were elevated from 37% to 40% and 43%, respectively. On increasing the monomer concentration further to 0.45 mol·L^−1^, the yield was not improved. It can be inferred that the optimal monomer concentration is 0.38 mol·L^−1^.

#### 3.1.4. Optimization of CuCl_2_/TT Molar Ratios

The effect of the CuCl_2_/TT molar ratios on the polymerization yield is significant. As the CuCl_2_/TT molar ratios increased from 0.5 to 1.0, 3.0, and 4.0, the corresponding polymerization yields changed from 53% to 72%, 49%, and 46%, respectively. Few reaction active centers are generated at low CuCl_2_/TT molar ratios, whilst the growth of polymeric chains is easier to terminate with too high CuCl_2_/TT molar ratios, explaining why a good polymerization yield (72%) of PTT was obtained at a moderate CuCl_2_/TT molar ratio (1.0).

#### 3.1.5. Optimization of Polymerization Temperature

The effect of the reaction temperature on the yield of the polymer is also evident. On increasing the polymerization temperatures from 5 to 50 °C, the PTT polymers exhibited a maximal polymerization yield (72%) at 25 °C. Too high a temperature though induces violent chain termination, whilst too low a temperature can lead to a low polymerization rate and less monomer initiation, explaining the reason that a moderate temperature of 25 °C is beneficial to the synthesis of PTT.

### 3.2. Chemical Structure of PTT Nanoparticles

#### 3.2.1. UV-Vis Spectra

**Figure 1:**
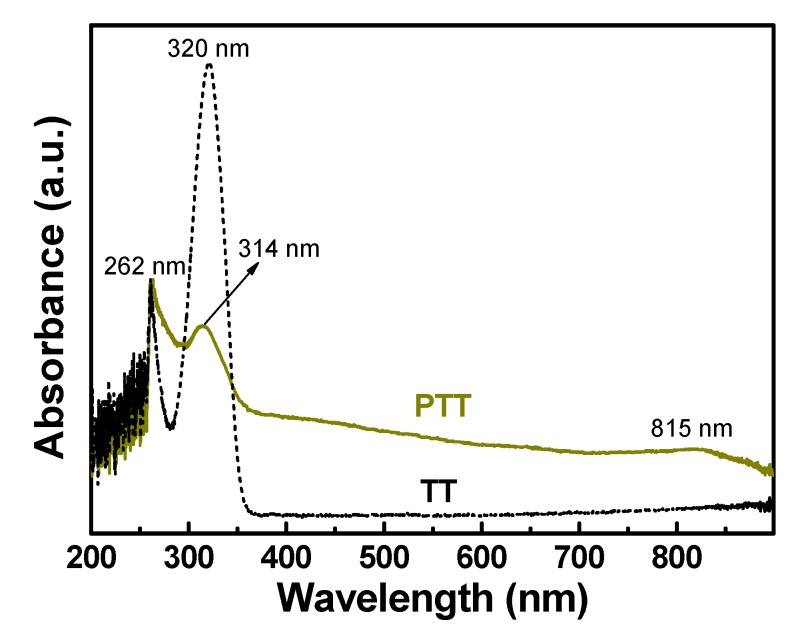
UV-Vis absorption spectra of the TT monomer and PTT polymer prepared with the CuCl_2_/TT molar ratio of 1.0 and the TT concentration of 0.38 mol·L^−1^ in dimethylformamide (DMF) at 25 °C for 24 h.

The UV-Vis spectral test solution was placed in a quartz cuvette. The UV-Vis spectra of TT monomer and PTT polymer are shown in Figure 1. The TT monomer exhibits two strong absorption peaks centered at 262 and 320 nm, assigning to the π→π* transition of the thiadiazole moiety and n→π* transition of the –S–(HS)C=N– moiety in the TT molecule, respectively [38,39]. By contrast, the spectrum of PTT polymer shows three peaks centered at 262, 314, and 815 nm. The new broad absorption at 815 nm is attributed to an intramolecular charge transfer of the entire macromolecules [40]. Summarizing, the difference in UV-Vis absorptions of polymer and monomer demonstrates that the polymerization of TT monomers indeed occurred. 

#### 3.2.2. FT-IR Spectra

**Figure 2:**
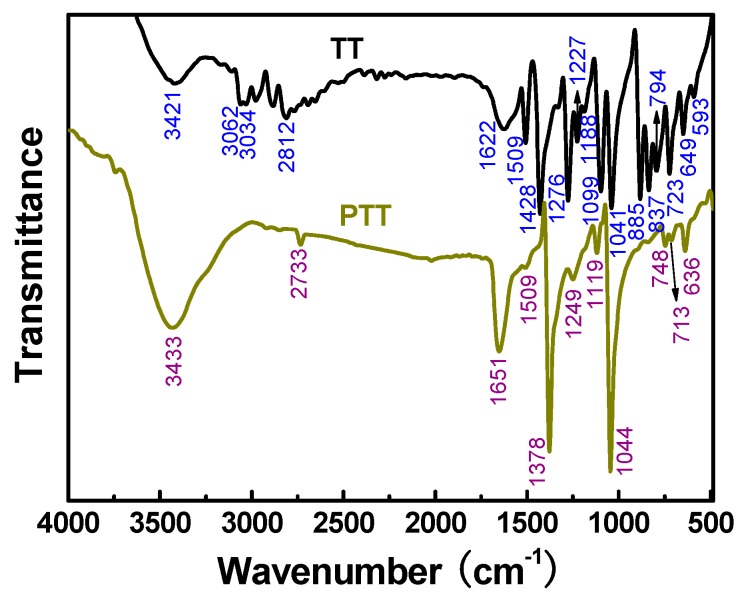
Fourier transform infrared (FT-IR) spectra of the TT monomer and PTT polymer prepared with a CuCl_2_/TT molar ratio of 1.0 and TT concentration of 0.38 mol·L^−1^ in DMF at 25 °C for 24 h.

The FT-IR spectra of the TT monomer and PTT polymer are shown in Figure 2 and the corresponding absorption assignments are summarized in Supplementary Materials Table S1. The TT monomer showed a weak absorption band at 2812 cm^−1^ and a shoulder peak at 3034–3062 cm^−1^ corresponding to the symmetric stretching of the –SH group and C–H bond on the thiadiazole ring [6,41,42]. The former band became much weaker and shifted to lower wavenumber (2733 cm^−1^) while the latter shoulder peak vanished in the spectrum of PTT polymer, indicating that the –SH and C–H groups took part in the polymerization. The bands at 649, 1041, and 1622 cm^−1^, appearing in the spectrum of TT monomer, belong to the stretching of C–S–C, N–N, and C=N bonds [6,41], respectively. For PTT polymer, these three bands exhibited a slight shift in wavenumber and become obviously more intense after the TT monomers were polymerized because the formation of macromolecular chains changed the electron cloud density of each group. Besides, the FT-IR spectrum of TT monomer showed characteristic bands at 1227, 1276, and 1428 cm^−1^ ascribed to the C–N stretching, thioamide II mode and N–H deformation [7], whilst these bands weakened and shifted slightly in position or vanished in the FT-IR spectrum of PTT polymer because the thione tautomer of the monomer was basically inhibited after the formation of macromolecular chains. In addition, a new and strong band at 1378 cm^−1^ appeared in the FT-IR spectrum of PTT polymer because the formation of macromolecular chains enhances the ring vibration absorption. After all, the vibration absorption band at 520–537 cm^−1^ which represents the formation of S–S bonds according to previous reports [7,8,12], did not appear in the FT-IR spectrum of PTT polymer. Thus, it is reasonable to infer that the chemical oxidative polymerization of TT occurs most likely through the head-to-tail coupling between the oxidizable C(5)–H and S(2)–H groups.

#### 3.2.3. XPS Spectra

The high-resolution XPS spectra of C 1s, N 1s, and S 2p for TT monomer and PTT polymer are shown in Supplementary Materials Figure S2. The C 1s spectrum of TT monomer was deconvoluted into three component peaks at 284.81, 286.31, and 288.76 eV ascribed to adventitious carbon (C–C/C=C), C–N/C=N, and C–S/C=S, respectively (Supplementary Materials Figure S2a) [43]. The PTT polymer, however, exhibited an obviously higher atomic concentration of C–S/C=S bonds (Supplementary Materials Figure S2b), which proves the formation of additional carbon–sulfur bonds. The N 1s spectrum of TT showed two component peaks at 398.80 and 400.56 eV due to C–N and –N=/N–N bonds [9,44]. By contrast, the peak at 398.80 eV vanished and a new peak at 401.30 eV (–N^+^H–) appeared in the N 1s spectrum of PTT, because the thione tautomerization of the monomer was basically inhibited after polymerization and the nitrogen atoms were protonated with hydrogen ions generated during the polymerization reaction. The S 2p region of TT monomer was decomposed into two groups of component peaks at 161.91/163.03 eV and 163.95/165.07 eV, which were ascribed to S–H or exocyclic sulfur, and heterocyclic sulfur (C–S and C=S), respectively. By contrast, as can be seen from the S 2p spectrum of PTT polymer (Supplementary Materials Figure S2b), the four peaks shifted positively by 0.56–0.81 eV. Further, the atomic concentration of S–H/exocyclic sulfur (–S–) groups in PTT polymer increased by about 13% in comparison with that in TT monomer, indicating that S–H groups were converted to exocyclic sulfur (–S–) moieties. These results confirm that the coupling of TT monomers occurs through the head-to-tail linkage between C(5) and S(2) sites, basically coinciding with the FT-IR spectral results.

#### 3.2.4. Wide Angle X-Ray Diffraction

**Figure 3:**
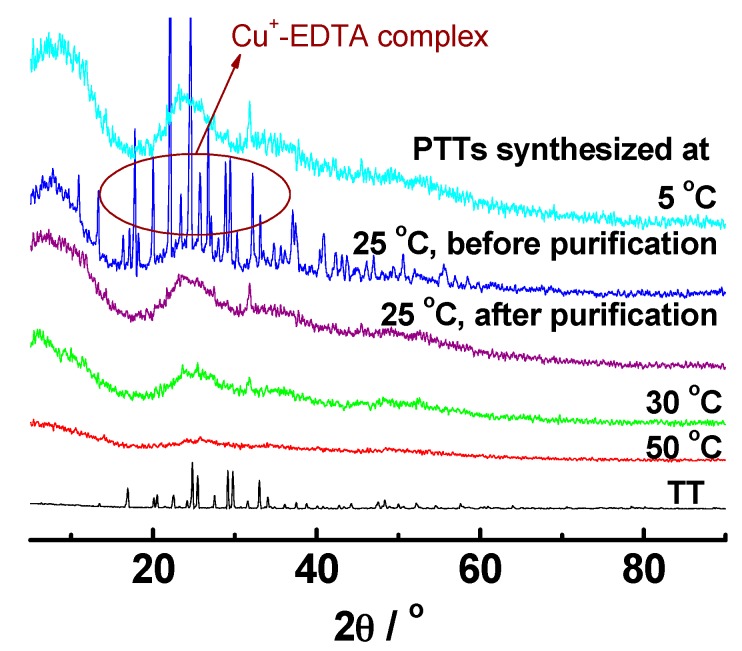
Wide-angle X-ray diffraction (WAXD) diffractograms of the TT monomer and PTT polymers prepared with a CuCl_2_/TT molar ratio of 1.0 and a TT concentration of 0.38 mol·L^−1^ in DMF for 24 h.

The wide-angle X-ray diffraction patterns of TT monomer and PTT polymers synthesized at different polymerization temperatures are illustrated in Figure 3. All of these PTT polymers displayed a broad and diffuse diffraction peak in the range from 5° to 30°, which is an indication of amorphous structures. It appears that the PTTs synthesized at lower temperatures had slightly higher crystallinity. Note that, the removal of by-product CuCl entrapped in the polymers is a tricky problem because CuCl is insoluble in acid water, alkaline water, pure water, and general organic solvents. We used EDTA as a complexing agent to convert cuprous ions into soluble complex Cu^+^-EDTA, which could be removed by washing with excess water and ethanol (See Figure 3). By contrast, the highly crystalline TT monomer showed many sharp diffraction peaks in the range of 16–36°. The amorphous structures are favorable for the penetration and then adsorption of heavy metal ions into the PTT nanoparticles [45].

#### 3.2.5. MALDI/TOF Mass Spectra

**Figure 4:**
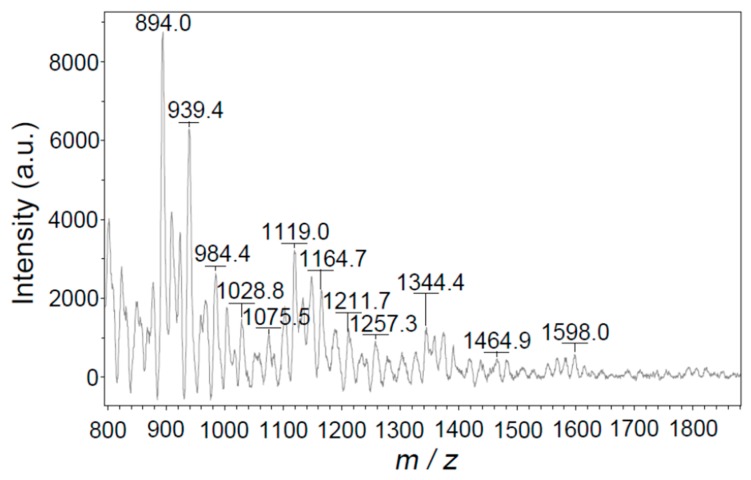
Matrix-assisted laser desorption/ionization/time-of-flight (MALDI/TOF) mass spectrum of tetrahydrofuran (THF) soluble part of PTT prepared with a CuCl_2_/TT molar ratio of 1.0 and a TT concentration of 0.38 mol·L^−1^ in DMF at 25 °C for 24 h.

The molecular weights of PTT were estimated by MALDI/TOF mass spectrometry (Figure 4) and the proposed compositions regarding each quasi-molecular ion are summarized in Supplementary Materials Table S2. It was found that the molecular weights of THF soluble part of PTT polymer are mostly in the range of 894–1598 corresponding to the polymerization degree of 7–13. This implies that the THF soluble part of PTT has a wide polymer chain length distribution. It is surprisingly to note that the actual polymerization degree cannot be simply calculated as *m*/*z* value divided by the mass of a BT monomer unit (–C_2_N_2_S_2_–, *m* = 116.15). The calculated mass differential (Δ*m*/*z*) between some neighboring quasi-molecular ions, is about 43.5–47, a value approximately equivalent to two times the mass of Na^+^ (22.99). This reveals that the PTT molecules formed metal ion adducts with Na^+^. Since the –S–, –SH, N–N and =N– groups on the PTT molecules have a strong affinity toward H^+^, Na^+^, K^+^ or other cations from the dithranol matrix, THF solvent or glassware, the sulfur and nitrogen atoms are sometimes ionized by these ions in order to form quasi-molecule ions or metal ion adducts (See Supplementary Materials Table S2) [45,46]. Therefore, the quasi-molecule ions or metal ion adducts can be attributed to the composition as expressed in Formula (1):

[H(C_2_N_2_S_2_)*_n_*H +*a*K +*b*Na +*c*H]^+^ (*n* ≥ 2; *a*, *b*, *c* = 0, 1, 2, 3, …) (1)

As can be seen from Supplementary Materials Table S2, the calculated values of *m*/*z* for the proposed compositions coincide well with the experimental values of *m*/*z*, suggesting the rationality of the proposed compositions as expressed in Formula (1). After all, our solubility experiments showed that PTT is insoluble in deionized water, methanol, ethanol, 1 mol·L^−1^ HCl aqueous solution, and only a small part (about 20 wt %) of the PTT can be dissolved in DMF, NMP, THF, and other strong solvents. The molecular weights of the insoluble parts can be measured neither by MALDI/TOF mass spectrometry nor by the high temperature gel permeation chromatography (HT-GPC) method. Presumably, the insoluble parts have much higher molecular weights than the soluble parts.

### 3.3. Polymerization Mechanism of PTT

To further reveal the PTT structure and related polymerization mechanism, we calculated the frontier orbit proportion and the electron spin density of each atom for the TT monomer (See Supplementary Materials Figure S1) at a B3LYP/6-31G (*d*) level by using Gaussian 09 software (Supplementary Materials Tables S3 and S4). For TT monomer, the highest occupied molecular orbital (HOMO) proportions of atoms (Supplementary Materials Table S3) are sorted in the following order: S(2) > N(3) > C(5) > C(2) > N(4) > S(1). In other words, S(2), N(3), and C(5) atoms of TT monomer are more active in the polymerization reaction. Since the reaction between the activated molecules takes place mostly on the frontier molecular orbitals and their adjacent orbitals and the N(3) atom is not linked to an active hydrogen, it can be inferred that the dehydro-coupling polymerization of TT occurs preferentially at the S(2) and C(5) positions. In addition, electron spin density (ESD) could be another decisive factor in judging the possibility of dehydro-coupling occurring [47,48]. The ESD of TT monomer as a radical cation was calculated at the B3LYP/6-31G (*d*) level, as summarized in Supplementary Materials Table S4. The results suggest that S(2) and C(5) atoms possess the highest and second highest ESD respectively, further implying the occurrence of S(2)–C(5) coupling during polymerization.

**Scheme 2:**
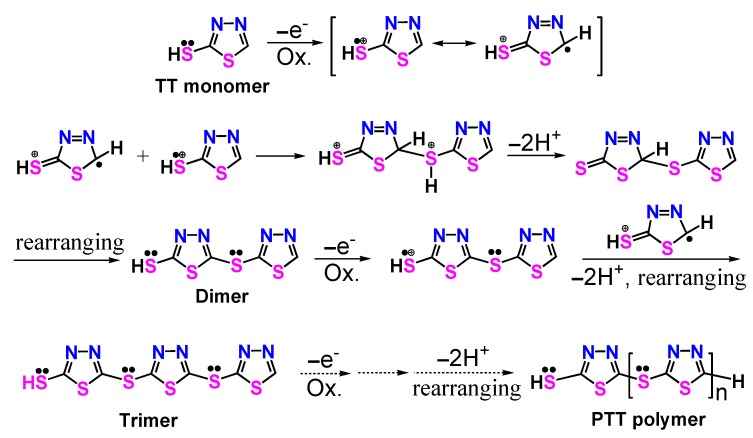
Proposed polymerization mechanism of TT.

In view of FT-IR, XPS, and MALDI/TOF mass analyses as well as theoretical calculations, one could logically draw a conclusion that the chemical oxidative dehydrogenation polymerization of TT occurs most probably by head-to-tail coupling, i.e., S(2)–C(5) coupling between S(2)–H and C(5)–H groups. Accordingly, a polymerization mechanism of TT can be proposed in Scheme 2. Firstly, the mercapto groups of TT were oxidized to generate radical cations. These unstable radical cations then rapidly coupled with each other to produce a dimer with the consumption of two protons and subsequent ring rearrangement. The dimer was more easily oxidized than the monomer to form dimer radical cations, which could couple with monomer radical cations or themselves to produce trimer or tetramer. The polymerization reaction was further propagated to form long PTT polymer chains.

### 3.4. Morphology of PTT Nanoparticles

**Figure 5:**
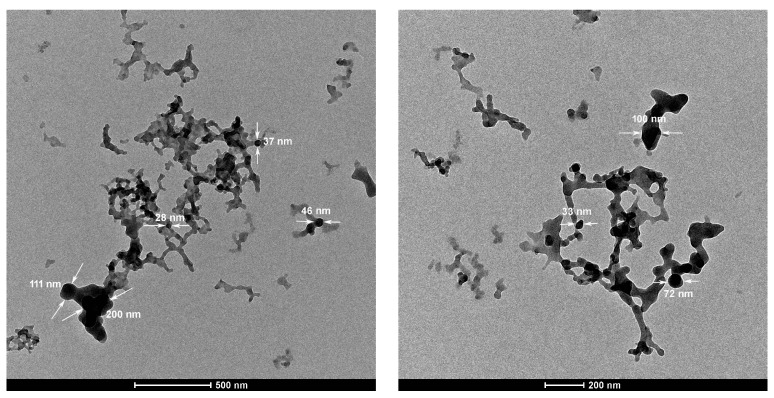
Transmission electron microscopy (TEM) images of the PTT prepared with a CuCl_2_/TT molar ratio of 1.0 and a TT concentration of 0.38 mol·L^−1^ in DMF at 25 °C for 24 h.

The morphology of PTT was analyzed by TEM as shown in Figure 5. The as-formed PTT polymer looks like uniform nanoparticles with a diameter of 30–200 nm although there is a slight deformation of the morphology of PTT nanoparticles under the high-energy electron beam of TEM, suggesting some flexibility of PTT molecular chains. The formation of these stable nanoparticles is due to electrostatic repulsion on the surface of as-formed nanoparticles, which avoids secondary growth or overgrowth of polymers stacked on the initially formed nanoparticles [45]. 

### 3.5. Adsorption of Heavy Metal Ions

#### 3.5.1. Adsorption Capacity

The PTT nanoparticles display a powerful adsorbability toward heavy metal ions due to the presence of a large number of –S–, –SH, N–N and =N– groups containing lone pairs of electrons on the PTT macromolecular chains verified by the FT-IR spectroscopy and MALDI/TOF mass. To investigate the adsorption capacity of PTT nanoparticles, ten kinds of heavy metal ions such as Ag^+^, Hg^2+^, Zn^2+^, Cd^2+^, Ni^2+^, Cr^3+^, Pb^2+^, Cu^2+^, Fe^3+,^ and Co^2+^ at the same initial concentration of 200 mg·L^−1^ were used separately for adsorption testing, and the experiments were conducted by batch test. The results are summarized in Table 1, from which one can find that the PTT nanoparticles exhibited excellent adsorption capacity (*Q*) for most of the metal ions and the *Q* values followed the order: *Q*(Ag^+^) > *Q*(Hg^2+^) > *Q*(Pb^2+^) > *Q*(Fe^3+^) > Q(Zn^2+^) > *Q*(Cd^2+^) > *Q*(Cu^2+^) > *Q*(Ni^2+^) > *Q*(Cr^3+^) > *Q*(Co^2+^). The highest and second highest adsorption capacities were up to 193.1 mg (Ag)·g^−1^ and 186.9 mg (Hg)·g^−1^ respectively, corresponding to adsorption ratios of 96.8% for Ag^+^ and 94.8% for Hg^2+^, respectively. The polyfunctional groups including –S–, –SH, N–N, and =N– could efficiently combine metal ions by means of sharing lone pairs of electrons to form chelating bonds (Scheme 1), which explains the reason that the PTT nanoparticles exhibited such good adsorption capacities.

#### 3.5.2. Adsorption Selectivity

As summarized in Table 1, the theoretical selectivity coefficients of Ag^+^ and Hg^2+^ ions versus the other eight ions range from 2.65 to 19.51, signifying that coexisting metal ions may not greatly influence the robust adsorbability of PTT nanoparticles toward Ag^+^ and Hg^2+^ ions. In the competitive adsorption system (Table 2), the adsorption ratio (*q*, %) order onto the nanoparticles was Hg^2+^ > Ag^+^ > Fe^3+^ > Cu^2+^ > Co^2+^ > Pb^2+^> Cd^2+^ > Zn^2+^ > Cr^3+^ > Ni^2+^ when each initial metal ion concentration was 20 mg·L^−1^ in the mixed solution. The PTT nanoparticles exhibited the highest adsorptivity for Hg^2+^ (86.6%) and the second highest adsorptivity for Ag^+^ (75.5%), and the adsorptivity for the other eight metal ions was in the range of 35.0%–66.0%. This indicates that the most powerful adsorbability of Hg^2+^ and Ag^+^ ions onto the nanoparticles actually were slightly affected by eight coexisting metal ions. Since the –S–, –SH, N–N, and =N– groups on the polymer chains belong to soft bases, these soft bases preferentially adsorb the soft acids such as Hg^2+^ and Ag^+^ ions. On the other hand, the electron-rich features of these groups give them strong adsorbability for various metal ions. 

#### 3.5.3. Effect of pH on Metal Adsorption

**Figure 6:**
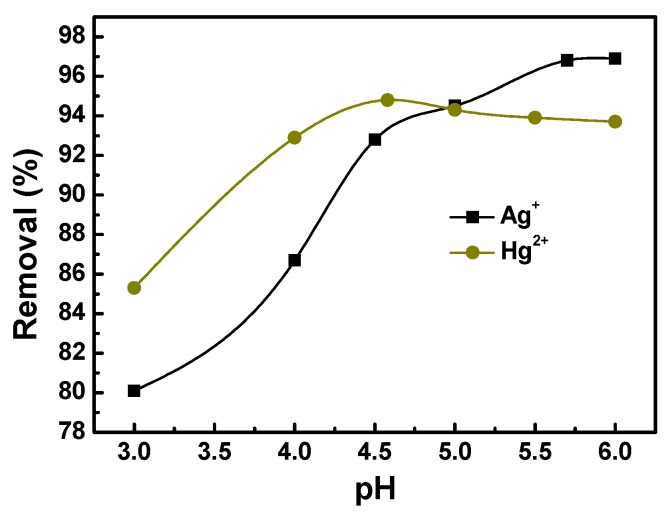
Effect of pH on the removal of Ag^+^ and Hg^2+^ ions onto PTT nanoparticles (initial metal concentration 200 mg·L^−1^; adsorbent dosage 1.0 g·L^−1^; contact time 2 h; shaking rate 200 rpm; 25 °C).

The effect of the pH of the medium on the removal of metal ions was examined by a batch technique. The pH of the medium was adjusted with 0.1 mol·L^−1^ HCl and 0.1 mol·L^−1^ NaOH. Since silver and mercury ions begin to precipitate as respective silver hydroxide and mercury hydroxide when the pH is over 6.0 [35,49], the range was restricted below a pH of 6.0. Fifty milligrams of PTT was added into 50 mL of silver nitrate or mercury nitrate solution (200 mg·L^−1^) and the mixture was oscillated at 25 °C for 2 h. After centrifugation, the mixture was separated into a supernatant and a precipitate and we got the Ag^+^ or Hg^2+^ concentration from the supernatant. As indicated in Figure 6, the optimum pH values for silver and mercury removal were 5.7–6.0 and 4.6, respectively. All the following adsorption experiments were carried out at these optimum pH values. At low pH, many of the sulfur and nitrogen atoms on the PTT molecular chains were protonated, and thus the complexation between these atoms and silver or mercury ions became weak [26]. When the pH was increased, the charge of metal species changed from cationic one to neutral or even anionic one. Thus, the removal ratio of metal ions began to decrease when the pH was increased to some extent [49].

#### 3.5.4. Adsorption Kinetics

**Figure 7:**
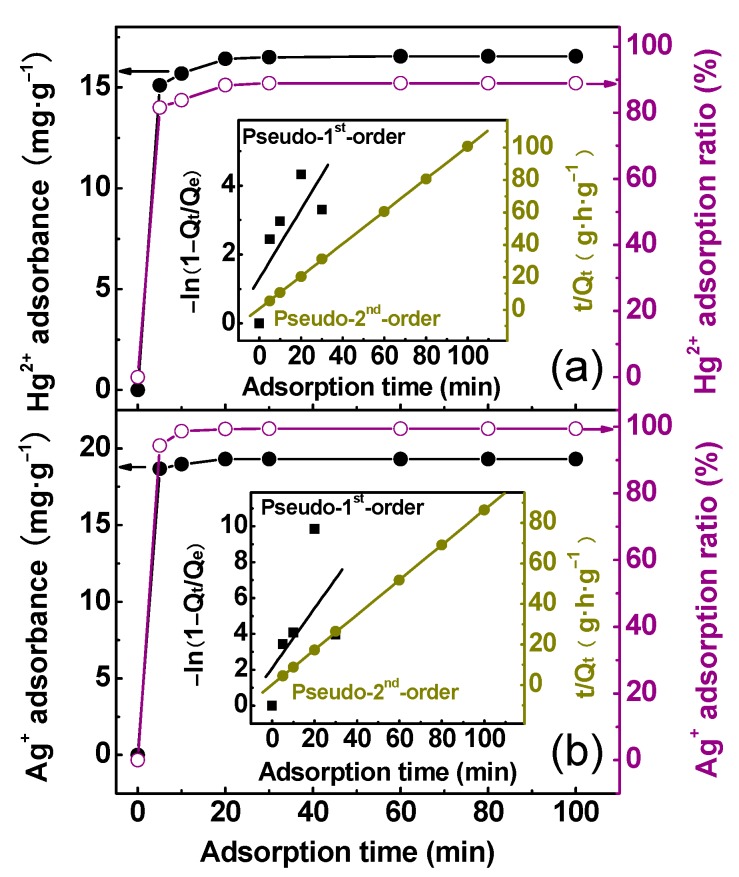
Adsorption kinetics of (**a**) Hg^2+^ and (**b**) Ag^+^ ions onto PTT nanoparticles at 25 °C in 50 mL of Hg(NO_3_)_2_ and AgNO_3_ solution at the same initial Hg^2+^ and Ag^+^ concentration of 20 mg·L^−1^ with 50 mg of adsorbent. Inset: kinetic model plots of the adsorption of Hg^2+^ and Ag^+^ onto the nanoparticles.

The adsorption kinetics of PTT was studied using Ag^+^ and Hg^2+^ as the target analytes. The rate at which adsorption occurs is of most importance when the adsorbent is employed for actual sewage treatment. Accordingly, it is important to establish the adsorption time dependence of such adsorbent adsorbing Hg^2+^ and Ag^+^ ions. Figure 7 shows the change in the adsorption of Hg^2+^ and Ag^+^ by the nanoparticles as a function of adsorption time at initial metal concentration of 20 mg·L^−1^. It could be seen that about 91.3% of the maximum adsorption of Hg^2+^ and 96.8% of the maximum adsorption of Ag^+^ could be achieved within only 5 min. The kinetic curves for Hg^2+^ and Ag^+^ ions indicated that the adsorption was very rapid and could reach equilibrium after about 20 min.

To reveal the kinetic mechanism, pseudo-first-order and pseudo-second-order models shown as Equations (2) and (3) were employed to interpret the adsorption process [50,51]. A good correlation of the kinetic data can be an indication of a certain adsorption mechanism.

The pseudo-first-order equation is expressed as

−ln(1 − *Q_t_*/*Q_e_*) = *k*_1_·*t* (2)

where *Q_e_* (mg·g^−1^) represents the adsorption capacity at equilibrium, *Q_t_* (mg·g^−1^) represents the adsorption capacity at time *t* (h), and *k*_1_ (h^−1^) denotes the adsorption rate constant for pseudo-first-order.

The pseudo-second-order equation can be represented by

*t*/*Q_t_* = *t*/*Q_e_* + 1/*h*_0_ = *t*/*Q_e_* + 1/(*k*_2_·*Q_e_*^2^) (3)

where *k*_2_ (g·mg^−1^·h^−1^) is the adsorption rate constant for pseudo-second-order, and *h*_0_ (mg·g^−1^·h^−1^) represents the initial adsorption rate of the pseudo-second-order kinetic equation.

The kinetic parameters for adsorption of Ag^+^ and Hg^2+^ ions by PTT nanoparticles are listed in Table 3. Based on the obtained correlation coefficients, the adsorptions of Ag^+^ and Hg^2+^ on PTT are both perfectly fit the pseudo-second-order model instead of the pseudo-first-order one, implying the main adsorption mechanism is chemical [52]. Besides, the experimental and theoretical values of *Q_e_* calculated from pseudo-second-order model coincide quite well, demonstrating the validity of that model to the adsorption systems under consideration. PTT is characterized by its high total molar content of nitrogen and sulfur, present in the form of –S–, –SH, N–N, and =N– groups which could be responsible for the metal ion binding through a chelation mechanism.

#### 3.5.5. Adsorption Mechanism 

**Figure 8:**
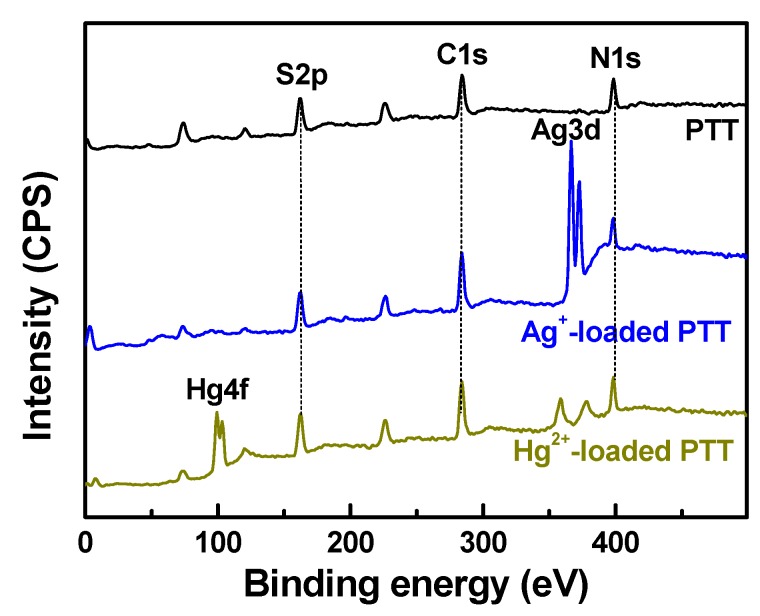
X-ray photoelectron (XPS) wide scan spectra of PTT, Ag^+^-loaded PTT and Hg^2+^-loaded PTT.

**Figure 9:**
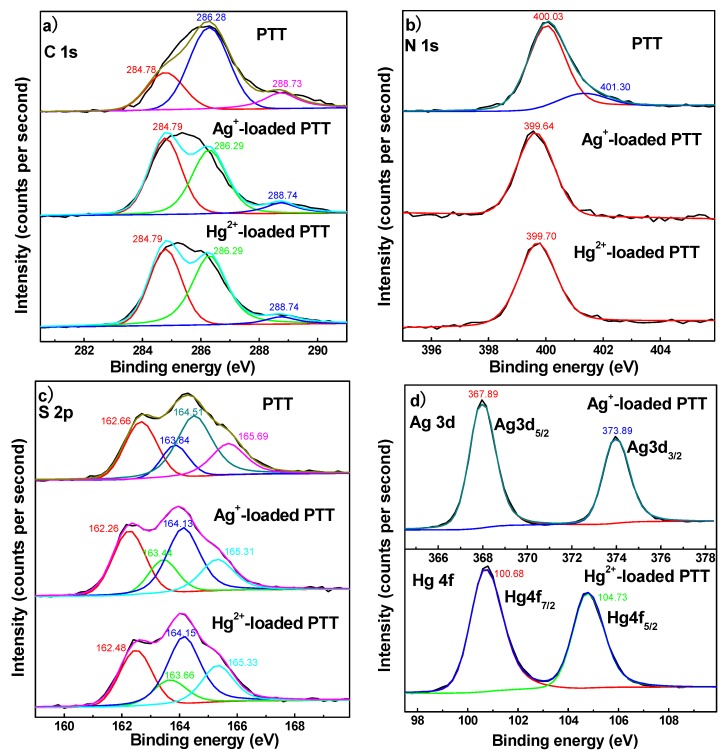
High-resolution XPS spectra of (**a**) C 1s, (**b**) N 1s, (**c**) S 2p, and (**d**) Ag 3d and Hg 4f.

The adsorption mechanisms of PTT nanoparticles for Ag^+^ and Hg^2+^ were further studied by XPS spectral analysis (Figure 8 and Figure 9). Several strong peaks with binding energies (BEs) of 162.0, 284.1, and 398.4 eV belong to S 2p, C 1s and N 1s orbitals, respectively, which exist in pristine PTT nanoparticles (Figure 8). Upon adsorbing Ag^+^ and Hg^2+^ ions, new shoulder peaks with BEs of approximately 366.4–372.7 eV and 99.2–103.1 eV appeared respectively, confirming that Ag^+^ and Hg^2+^ ions were adsorbed onto PTT nanoparticles. The high-resolution XPS spectra of C 1s, N 1s, S 2p, Ag 3d, and Hg 4f core levels are shown in Figure 9. All of the C 1s, N 1s, and S 2p regions were analyzed by peak deconvolution. The C 1s spectrum of PTT polymer was deconvoluted into three component peaks at 284.78, 286.28, and 288.73 eV, which were attributed to adventitious carbon (C–C/C=C), C–N/C=N, and C–S/C=S respectively (Figure 9a) [43]. The C 1s XPS of Ag^+^-loaded PTT, however, does not clearly show significant changes of BEs in comparison with that of PTT, indicating that the heterocyclic carbon in the PTT molecules does not contribute to the adsorption of Ag^+^ ion. The N 1s region of PTT was deconvoluted into two component peaks at 400.03 and 401.30 eV associated with –N=/N–N and –N^+^H–, respectively (Figure 9b) [9,44]. However, in the N 1s XPS of Ag^+^-loaded PTT, the former peak shifted to lower BE by 0.39 eV and the latter peak vanished, suggesting that nitrogen atoms took part in the adsorption of Ag^+^ ions. The negative shift of BE is due to a decrease in electron cloud density on the nitrogen atom once the Ag^+^–N coordination bonds were formed [53], and the reason for the disappearance of the peak at BE 401.30 eV is that the H^+^ ions on the –N^+^H– groups were replaced by Ag^+^ ions. The S 2p region of PTT was decomposed into two groups of component peaks at 162.66/163.84 eV and 164.51/165.69 eV, which are ascribed to S–H and exocyclic sulfur (–S–), and heterocyclic sulfur (C–S and C=S), respectively. By contrast, as can be seen from the S 2p spectrum of Ag^+^-loaded PTT (Figure 9c), all of these four peaks were shifted negatively to lower BEs by 0.38–0.40 eV, indicating that sulfur atoms also contributed to the adsorption of Ag^+^ ions.

The high-resolution XPS spectra of C 1s, N 1s, and S 2p for Hg^2+^-loaded PTT were similar to those for Ag^+^-loaded PTT, implying that the nitrogen and sulfur atoms on polymer chains also contributed to the adsorption of Hg^2+^ ions. In addition, both Ag 3d and Hg 4f XPS spectra (Figure 9d) exhibited two peaks at 367.89 and 373.89 eV assigned to Ag 3d_5/2_ and Ag 3d_3/2_; as well as 100.68 and 104.73 eV due to Hg 4f_7/2_ and Hg 4f_5/2_, respectively. The presence of a Ag 3d_5/2_ peak at 367.89 eV indicated that Ag^+^ formed a complex with –S–, –SH, N–N, and =N– bonds [26]. While the presence of Hg 4f_7/2_ peak at 100.68 eV implied that Hg^2+^ should form a complex with –S–, –SH, N–N, and =N– bonds [25].

In addition, pH changes in metal ion solutions before and after adding PTT nanoparticles were monitored so as to understand the adsorption mechanism better. After adsorption, the pH of Ag^+^ and Hg^2+^ solutions obviously declined. In fact, the pH of the Ag^+^ solution and Hg^2+^ solution clearly decreased by 0.33 (5.70 vs. 5.37) and 0.27 (4.58 vs. 4.31) respectively after being treated by the nanoparticles, probably because of the release of H^+^ ions by means of the ion exchanges, further implying the occurrence of ion exchanges. Thus, ion exchange could be another possible adsorption mechanism. However, it was a minor one, since the available H^+^ numbers on the polymer chains were after all limited [54].

#### 3.5.6. Comparison of Ag^+^ and Hg^2+^ Adsorbability with Other Adsorbents

On the basis of a careful comparison of the Ag^+^ and Hg^2+^ adsorbability onto several typical adsorbents including inorganics (such as carbon, silica and Fe_3_O_4_), natural bio-polymers (such as chitosan, cellulose, yeast, and wool), organic polymers, and inorganic-organic composites summarized in Table 4, the PTT nanoparticles have an even stronger adsorbability toward Ag^+^ and Hg^2+^ ions than most of the other adsorbents reported hitherto to the best of our knowledge. The Ag^+^ adsorption capacity of PTT is about 5–7 times that of activated carbon [30], cellulose nanocrystals [55], and waste yeast [56] and 2.4 times that of silica nanoparticles modified with trithiocyanuric acid [21], and is still obviously superior to that of others [26,34,36,57]. The PTT nanoparticles also exhibit much higher Hg^2+^ adsorption capacity than poly(hydroxyethylmethacrylate/chitosan) membranes [58] and poly(1-amino-5-chloroanthraquinone) [18], and have still obviously higher Hg^2+^ adsorption capacity when compared with others [33,35,59,60,61]. The fact that the PTT nanoparticles hold strong adsorbability for treating concentrated Ag^+^ and Hg^2+^ solutions of up to 200 mg·L^−1^ hints that the PTT nanoparticles will display very high removal capability and long durability if treating low-concentration silver or mercury-containing wastewater. Further, the removal speeds of Ag^+^ and Hg^2+^ by the PTT nanoparticles are both the most rapid so far. The PTT nanoparticles can achieve equilibrium adsorption in 200 mg·L^−1^ Ag^+^ and Hg^2+^ solutions both within 0.33 h at a very fast initial adsorption rate of up to 5364 and 1500 mg·g^−1^·h^−1^, respectively. By contrast, most of the other adsorbents could complete equilibrium adsorption in 0.5–48 h at a much slower initial adsorption speed. Presumably, the satisfactory Ag^+^ and Hg^2+^ adsorbability could be ascribed to an optimal synergic combination of the four kinds of active functional groups with the small particle size of the PTT nanoparticles.

#### 3.5.7. Desorption Studies and Regeneration of PTT

For the purpose of potential practical applications, it is of crucial importance to estimate the possibility of desorbing the metal ions adsorbed on PTT and reusing it. Inorganic acid and EDTA aqueous solutions have been generally used as desorbents to desorb heavy metal ions from adsorbents [33,62]. In this study, both 0.1 mol·L^−1^ EDTA and 1 mol·L^−1^ HNO_3_ aqueous solutions were used as an eluent to desorb the Ag^+^ and Hg^2+^ ions adsorbed on PTT nanoparticles and this process was repeated for five adsorption-desorption cycles. The desorption efficiency for five cycles is summarized in Table 5. The desorption efficiency did not obviously decline when the PTT regeneration cycles were carried out, indicating that both 0.1 mol·L^−1^ EDTA and 1 mol·L^−1^ HNO_3_ could efficiently desorb the Ag^+^ and Hg^2+^ ions adsorbed on PTT nanoparticles and no obvious loss in efficiency was observed even after five cycles. However, the desorption efficiency of EDTA solution is somewhat higher than that of HNO_3_ solution owing to the different mechanisms of these two eluents. EDTA can form stable complex with metal ions whereas the desorption in HNO_3_ solution could be attributed to the ion exchange mechanism. The EDTA solution is thus more effective in desorption than the HNO_3_ solution. Furthermore, the adsorption capacity of the PTT could still be maintained at over 91% level at the fifth cycle, as shown in Table 5. Consequently, the PTT nano-sorbent could be used repeatedly without significantly losing the adsorption capacity for Ag^+^ and Hg^2+^ ions.

## 4. Conclusions

Poly-2-mercapto-1,3,4-thiadiazole (PTT) nanoparticles were synthesized via a chemical oxidative dehydrogenation polymerization under mild conditions. The as-formed PTT polymers possess several unique features: facile one-pot synthesis; mild synthesis conditions (oxidant, CuCl_2_; medium, DMF; reaction temperature, 25 °C), and high total molar content of sulfur and nitrogen (>50%). The oxidant species, polymerization medium, TT monomer concentration, CuCl_2_/TT molar ratios, and polymerization temperature were thoroughly optimized for the formation of uniform PTT nanoparticles with a satisfactory polymerization yield and strong heavy metal ion adsorbability. The UV-Vis, FT-IR, XPS, and MALDI-TOF mass spectra analyses and theoretical calculations show that macromolecular chains of PTT were created via head-to-tail coupling, i.e., S(2)–C(5) coupling between S(2)–H and C(5)–H groups of the TT monomers. TEM observation showed that the as-formed PTT polymer was of nanoparticles with a diameter of 30–200 nm. The nano-scale and sulfur/nitrogen-rich features endow this polymer with wonderful heavy metal ion adsorbability. The PTT nanoparticles can be excellent adsorbents for mercury and silver ions, with an adsorption capacity as high as 186.9–193.1 mg·g^−1^ and a very short equilibrium adsorption time as low as 0.33 h, which makes them very competitive with most good Hg^2+^ and Ag^+^ sorbents previously reported. The adsorption kinetics followed the pseudo-second-order model, suggesting a major chemical adsorption. Regeneration of the nanoparticles can be achieved by using 0.1 mol·L^−1^ EDTA and 1 mol·L^−1^ HNO_3_ with an efficiency greater than 80%. It is expected that PTT nanoparticles would be a promising adsorbent for rapid removal of heavy metals and recovery of precious metals from aqueous solutions.

## Figures and Tables

**Table 1:** Adsorption capacity and adsorption ratio of ten metal ions with an initial concentration of 200 mg·L^−1^ for each metal ion at 25 °C for 24 h with a poly-2-mercapto-1,3,4-thiadiazole (PTT) sorbent dosage of 1.0 g·L^−1^.

Metal Ion Solutions	Adsorption Capacity (mg·g^−1^)	Adsorption Ratio (%)	Theoretical Selectivity Coefficient	
			Ag^+^	Hg^2+^
AgNO_3_	193.1	96.8	1.00	0.97
Hg(NO_3_)_2_	186.9	94.8	1.03	1.00
Zn(NO_3_)_2_	24.8	12.4	7.79	7.54
Cd(NO_3_)_2_	24.5	12.3	7.88	7.63
Ni(NO_3_)_2_	12.0	6.0	16.09	15.58
Cr(NO_3_)_3_	10.1	5.1	19.12	18.50
Pb(NO_3_)_2_	70.6	35.9	2.74	2.65
Cu(NO_3_)_2_	15.6	7.9	12.38	11.98
Fe(NO_3_)_3_	27.6	13.8	7.00	6.77
Co(NO_3_)_2_	9.9	5.2	19.51	18.88

**Table 2:** Competitive adsorption of ten metal ions onto 100 mg of PTT in 50 mL solution with an initial concentration of 20 mg·L^−1^ for each metal ion at 25 °C for 24 h.

Mixed Solution	Metal Ions	Adsorption Capacity (mg·g^−1^)	Adsorption Ratio (%)
AgNO_3_	Ag^+^	7.6	75.5
Hg(NO_3_)_2_	Hg^2+^	8.7	86.6
Zn(NO_3_)_2_	Zn^2+^	5.6	55.5
Cd(NO_3_)_2_	Cd^2+^	5.8	58.0
Ni(NO_3_)_2_	Ni^2+^	3.5	35.0
Cr(NO_3_)_3_	Cr^3+^	5.4	53.5
Pb(NO_3_)_2_	Pb^2+^	5.9	59.0
Cu(NO_3_)_2_	Cu^2+^	6.4	64.0
Fe(NO_3_)_3_	Fe^3+^	6.6	66.0
Co(NO_3_)_2_	Co^2+^	6.1	60.5

**Table 3:** Kinetic model equations for Ag^+^ and Hg^2+^ adsorption on PTT nanoparticles.

Mathematical Model	Equation	*R^2^*	Standard Deviation	*k*_1_(h^−1^) *k*_2_(g·mg^−1^·h^−1^) *h*_0_(mg·g^−1^·h^−1^)	*Q_e_* Found by	
					Experiment	Equation
Ag^+^ adsorption						
Pseudo-1st-order	−ln(1 − *Q_t_*/*Q_e_*) = 9.9918*t* + 2.1009	0.3191	3.3821	*k*_1_ = 9.9918 *k*_2_ = 14.4009 *h*_0_ = 5.3642 × 10^3^	19.30	–
Pseudo-2nd-order	*t*/*Q_t_* = 0.05168*t* + 2.0793 × 10^−4^	0.99998	1.8462 × 10^−4^		19.30	19.35
Hg^2+^ adsorption						
Pseudo-1st-order	−ln(1 − *Q_t_*/*Q_e_*) = 5.9904*t* + 1.3080	0.5576	1.2366	*k*_1_ = 5.9904 *k*_2_ = 5.4834 *h*_0_ = 1.5001 × 10^3^	16.54	–
Pseudo-2nd-order	*t*/*Q_t_* = 0.06002*t* + 6.6663 × 10^−4^	0.9992	3.6159 × 10^−4^		16.54	16.66

**Table 4:** Comparison of equilibrium adsorption capacity (*Q_e_*), adsorption ratio (*q*), and equilibrium time (*T_Qe_*) of various adsorbents toward Ag^+^ and Hg^2+^.

Adsorbents	*C*_0_ (mg·L^−1^)	pH	*Q_e_* (mg·g^−1^)	*q* (%)	*T_Qe_* (h)
Ag^+^ adsorption					
PTT (this work)	200	5.7	193.1	96.8	0.33
Activated carbon [30]	120	4.5	38.8	97	5
Thiourea/glutaraldehyde grafted O-carboxymethyl chitosan [36]	160.5	5.0	156.32	97.4	48
Cellulose nanocrystals [55]	107.8	6.39	34.4	64	12
Diethylenetriamine-bridged polysilsesquioxanes [34]	539.3	–	162.9	30.2	12
NH_2_-MCM-41 nano porous adsorbent [57]	300	7	101.45	94.32	3
Waste yeast [56]	100	3	27.9	93	1
Silica nanoparticles modified with 3-amino-5-mercapto-1,2,4-triazole [26]	400	5	124.52	62.3	0.33
Silica nanoparticles modified with trithiocyanuric acid [21]	500	5.0	80	32	0.5
Hg^2+^ adsorption					
PTT (this work)	200	4.6	186.9	94.8	0.33
Poly(hydroxyethylmethacrylate/chitosan) membranes [58]	400	5.0	68.8	–	0.75
Poly(2-hydroxyethylmethacrylate) gel beads modified with poly(ethyleneimine) [32]	300	5.0	335	–	1.0
Dithiocarbamate-anchored polymer/organosmectite composites [59]	400	6.0	157.3	–	1.0
Fe_3_O_4_@Zn(btb) nanospheres [60]	200	8	129.87	64.93	0.5
Cross-linked magnetic chitosan-phenylthiourea [61]	200	5	135	67.50	3
Poly(1-amino-5-chloroanthraquinone) [18]	100.3	6	83.25	83	2
Wool-grafted-poly(cyano-acetic acid α-amino-acrylichydrazide) (wool-g-PCAH) chelating fibers [35]	100	5	154.32	93.3	1.5
Modified poly(ethylene terephthalate) chelating fibers [33]	150	5	120.02	80.01	0.83

**Table 5:** Desorption efficiencies (%) and adsorption capacities (%) of Ag^+^ and Hg^2+^ on PTT nanoparticles from five adsorption-desorption cycles.

Cycle Number	Desorption Percentage (%)				Adsorption Capacity (%)	
	EDTA Solution		HNO_3_ Solution		Ag^+^	Hg^2+^
	Ag^+^	Hg^2+^	Ag^+^	Hg^2+^		
1	98.4	99.2	96.5	97.4	100	100
2	95.9	97.8	91.7	92.2	97.2	97.6
3	94.1	95.3	90.3	91.8	96.3	96.8
4	92.6	93.1	85.9	86.5	94.4	95.7
5	87.2	89.5	80.1	82.6	91.5	92.3

## Supplementary Materials

The following are available online at http://www.mdpi.com/2073-4360/10/2/150/s1, Figure S1: Molecular model of TT monomer with minimized energy, Figure S2: High-resolution C 1s, N 1s and S 2p XPS spectra of (**a**) TT monomer and (**b**) PTT polymer prepared with the CuCl_2_/TT molar ratio of 1.0 and the TT concentration of 0.38 mol·L^−1^ in DMF at 25 °C for 24 h, Table S1: FT-IR spectrum data of solid TT and PTT and their assignments, Table S2: Proposed composition and corresponding theoretical mass-to-charge ratio of PTT molecules, Table S3: Main composition and proportion (%) of frontier orbitals in TT, Table S4: Main atomic electron spin densities for TT.
