# Supplementary material for: Facile Synthesis, Characterization of Poly-2-mercapto-1,3,4-thiadiazole Nanoparticles for Rapid Removal of Mercury and Silver Ions from Aqueous Solutions
Source: Polymers (Basel). 2018 Feb 6;10(2):150. doi: 10.3390/polym10020150 (PMC6415195; doi:10.3390/polym10020150)
Supplement: Supplementary file 1 [file polymers-10-00150-s001.docx]

**Supplementary Materials: Facile Synthesis, Characterization of Poly-2-mercapto-1,3,4-thiadiazole Nanoparticles for Rapid Removal of Mercury and Silver Ions from Aqueous Solutions**

Shaojun Huang ^1,^*, Chengzhang Ma ^2^, Chao Li ^2^, Chungang Min ^1^, Ping Du ^1^, Yi Xia ^1^, Chaofen Yang ^1^ and Qiuling Huang ^1^

^1^ Research Center for Analysis and Measurement, Kunming University of Science and Technology, Kunming 650093, China; minchungang@163.com (C.M.); dupin515@163.com (P.D.); xiayi0125@163.com (Y.X.); yangmlh@163.com (C.Y.); hql1975@eyou.com (Q.H.)

^2^ School of Materials Science and Engineering, Kunming University of Science and Technology, Kunming 650093, China; [mcz219@yeah.net](mailto:mcz219@yeah.net) (C.M.); lichao2527@yeah.net (C.L.)

***** Correspondence: [huangshaojun1975@163.com](mailto:huangshaojun1975@163.com) or sjhuang@kmust.edu.cn; Tel.: +86-0871-6511-9674


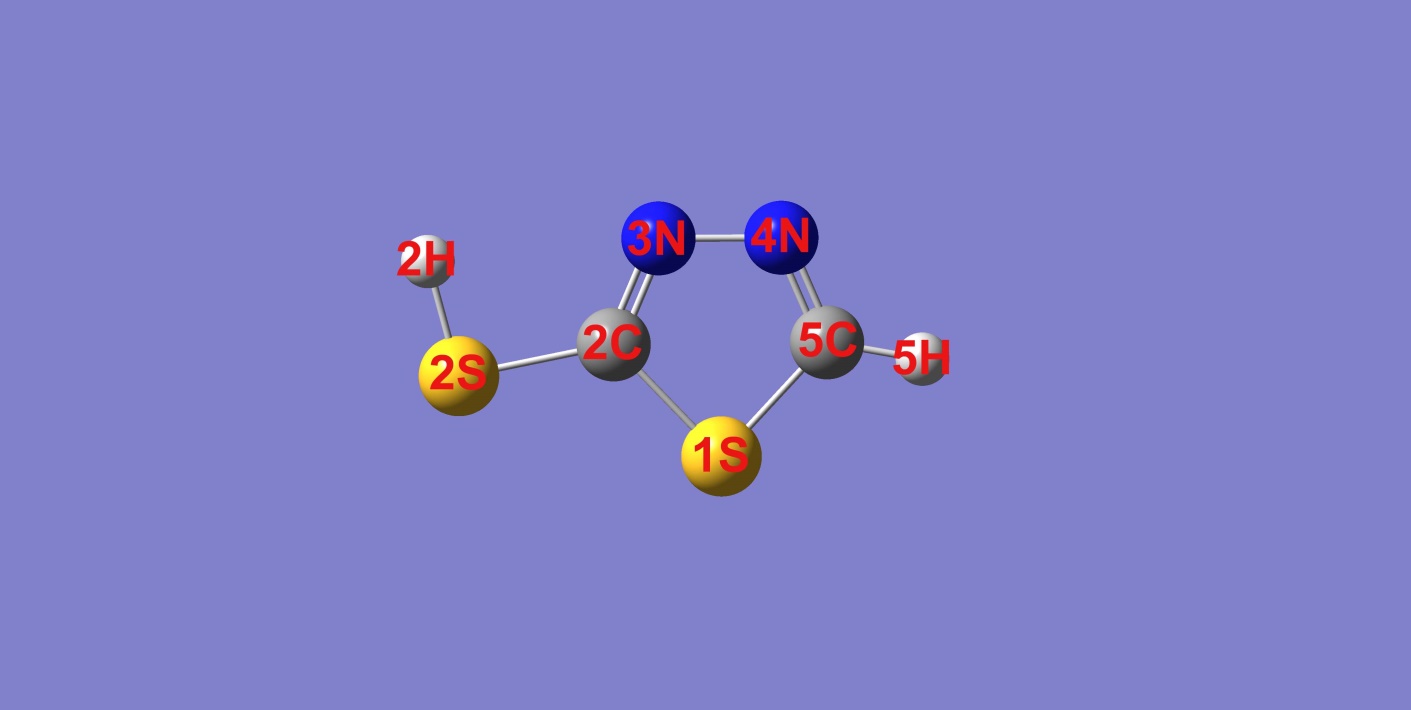


**Figure S1.** Molecular model of TT monomer with minimized energy.

**Figure S2.** High-resolution C 1s, N 1s and S 2p XPS spectra of (**a**) TT monomer and (**b**) PTT polymer prepared with the CuCl_2_/TT molar ratio of 1.0 and the TT concentration of
0.38 mol·L^−1^ in DMF at 25 ºC for 24 h.

**Table S1.** FT-IR spectrum data of solid TT and PTT and their assignments [1‒6].

| **IR data / cm^−1^ of solid TT** | **IR data / cm^−1^ of solid PTT** | **Assignment** |
| --- | --- | --- |
| 3421 (m) | 3433 (s) | υ (adventitious H_2_O) |
| 3062 (m), 3034 (m) | ‒ | υ (C–H) |
| 2812 (m) | 2733 (w) | υ _s._ (S–H) |
| 1622 (m) | 1651 (s) | υ _s._ (C=N) |
| 1509 (s) | 1509 (w) | δ (N–H)_ip._ |
| 1428 (vs) | ‒ | δ (N–H)_rock_ |
| ‒ | 1378 (vs) | υ (thiadiazole ring skeleton) |
| 1276 (vs) | 1249 (w) | thioamide II mode |
| 1227 (m) | ‒ | υ (C–N) |
| 1188 (w) | 1119 (w) | υ (thiadiazole ring skeleton) |
| 1099 (vs) | ‒ | υ (C=S) |
| 1041 (vs) | 1044 (vs) | υ (N–N) |
| 885 (vs), 837 (vs) | ‒ | δ (C–H)oop |
| 794 (m) | 748 (w) | δ (N–H)_tor._ |
| 732 (s) | 713 (w) | υ_as._ (C–S–C endocyclic) |
| 649 (m) | 636 (m) | υ_s._ (C–S–C endocyclic) |
| 593 (w) | ‒ | υ (S–C–S) |
|  |  |  |
| w: weak, m: medium, s: strong, vs: very strong, υ: stretch, δ: deformation, as.: asymmetric, s.: symmetric, ip.: in-plane, tor.: torsion, oop: out-of-plane | | |

**Table S2.** Proposed composition and corresponding theoretical mass-to-charge ratio of PTT molecules.

| **Experimental value of *m*/*z*** | **Proposed composition** | **Calculated value of *m*/*z*** |
| --- | --- | --- |
| 894.0 | [H(C_2_N_2_S_2_)_7_H +2K+H]^+^ | 894.3 |
| 939.4 | [H(C_2_N_2_S_2_)_8_H+8H]^+^ | 939.3 |
| 984.4 | [H(C_2_N_2_S_2_)_8_H +2Na+7H]^+^ | 984.3 |
| 1028.8 | [H(C_2_N_2_S_2_)_8_H +4Na+5H]^+^ | 1028.3 |
| 1075.5 | [H(C_2_N_2_S_2_)_9_H +Na+5H]^+^ | 1075.4 |
| 1119.0 | [H(C_2_N_2_S_2_)_9_H +3Na+3H]^+^ | 1119.4 |
| 1164.7 | [H(C_2_N_2_S_2_)_10_H +H]^+^ | 1164.5 |
| 1211.7 | [H(C_2_N_2_S_2_)_10_H +2Na+2H]^+^ | 1211.5 |
| 1257.3 | [H(C_2_N_2_S_2_)_10_H +4Na+2H]^+^ | 1257.5 |
| 1344.4 | [H(C_2_N_2_S_2_)_11_H+K +Na+3H]^+^ | 1344.8 |
| 1464.9 | [H(C_2_N_2_S_2_)_12_H +3Na]^+^ | 1464.8 |
| 1598.0 | [H(C_2_N_2_S_2_)_13_H+K +2Na+H]^+^ | 1598.1 |

**Table S3.** Main composition and proportion (%) of frontier orbitals in TT.

| **Atom** | **HOMO–1** | **HOMO** | **LUMO** | **LUMO+1** |
| --- | --- | --- | --- | --- |
| S(1) | 6.62 | 4.90 | 23.00 | 10.36 |
| C(2) | 13.28 | 7.41 | 31.11 | 65.84 |
| S(2) | 0.96 | 58.64 | 5.77 | 11.55 |
| N(3) | 33.31 | 11.94 | 5.98 | 1.84 |
| N(4) | 35.17 | 6.19 | 8.29 | 1.40 |
| C(5) | 10.27 | 10.92 | 25.85 | 8.37 |

**Table S4.** Main atomic electron spin densities for TT.

| **Atom** | **Electron spin density** | **Atom** | **Electron spin density** |
| --- | --- | --- | --- |
| S(1) | –0.004652 | C(2) | 0.040873 |
| S(2) | 0.569823 | N(3) | 0.166581 |
| N(4) | 0.067438 | C(5) | 0.18845 |

**References**

1. Aydogdu, G.; Gunendi, G.; Zeybek, D.K.; Zeybek, B.; Pekyardimci, S. A novel electrochemical DNA biosensor based on poly-(5-amino-2-mercapto-1,3,4-thiadiazole) modified glassy carbon electrode for the determination of nitrofurantoin. *Sensor. Actuat. B-Chem.* **2014**, *197*, 211–219.
2. Shouji, E.; Oyama, N. Examination of the cleavage and formation of the disulfide bond in poly[dithio-2,5-(1,3,4-thiadiazole)] by redox reaction. *J. Electroanal. Chem.* **1996**, *410*, 229–234.
3. Jin, L.F.; Wang, G.C.; Li, X.W.; Li, L.B. Poly(2,5-dimercapto-1,3,4-thiadiazole)/sulfonated graphene composite as cathode material for rechargeable lithium batteries. *J. Appl. Electrochem.* **2011**, *41*, 377–382.
4. Pope, J.M.; Sato, T.; Shoji, E.; Oyama, N.; White, K.C.; Buttry, D.A. Organosulfur/conducting polymer composite cathodes II. Spectroscopic determination of the protonation and oxidation states of 2,5-dimercapto-1,3,4-thiadiazole. *J. Electrochem. Soc.* **2002**, *149*, A939–A952.
5. Kalimuthu P, John S A. Simultaneous determination of epinephrine, uric acid and xanthine in the presence of ascorbic acid using an ultrathin polymer film of 5-amino-1,3,4-thiadiazole-2-thiol modified electrode. *Anal. Chim. Acta* **2009**, *647*, 97–103.
6. Zhao, Y.X.; Sun, X.Y. Spectrometric identification of organic molecular structures, 1^st^ ed.; Science Press: Beijing, China, 2003; pp. 373–388, ISBN 7-03-010866-3.
